# Cultivating scientific literacy and a sense of place through course‐based urban ecology research

**DOI:** 10.1002/ece3.8985

**Published:** 2022-06-02

**Authors:** Justin M. Valliere

**Affiliations:** ^1^ 14702 Department of Biology California State University Dominguez Hills Carson California USA

**Keywords:** active learning, culturally responsive teaching, experiential learning, living laboratory, remote learning, urban wildlife

## Abstract

Undergraduate research experiences have been shown to increase engagement, improve learning outcomes, and enhance career development for students in ecology. However, these opportunities may not be accessible to all students, and incorporating inquiry‐based research directly into undergraduate curricula may help overcome barriers to participation and improve representation and inclusion in the discipline. The shift to online instruction during the COVID‐19 pandemic has imposed even greater challenges for providing students with authentic research experiences, but the pandemic may also provide a unique opportunity for creative projects conducted remotely. In this paper, I describe a course‐based undergraduate research experience (CURE) designed for an upper‐level ecology course at California State University, Dominguez Hills during remote learning. The primary focus of student‐led research activities was to explore the potential impacts of the depopulation of campus during the pandemic on urban coyotes (*Canis latrans*), for which there were increased sightings reported during this time. Students conducted two research studies, including an evaluation of urban wildlife activity, behavior, and diversity using camera traps installed throughout campus and analysis of coyote diet using data from scat dissections. Students used the data they generated and information from literature reviews, class discussions, and meetings with experts to develop a coyote monitoring and management plan for our campus and create posters to educate the public. Using the campus as a living laboratory, I aimed to engage students in meaningful research while cultivating a sense of place, despite being online. Students’ research outcomes and responses to pre‐ and post‐course surveys highlight the benefits of projects that are anchored in place‐based education and emphasize the importance of ecological research for solving real‐world problems. CUREs focused on local urban ecosystems may be a powerful way for instructors to activate ecological knowledge and capitalize on the cultural strengths of students at urban universities.

## Graphical abstract

Incorporating authentic research experiences into undergraduate courses may have tremendous benefits for student learning and professional development in ecology, even when such activities are conducted during online learning. Anchoring such research projects in local urban ecosystems may be an especially powerful educational tool for students on urban university campuses.

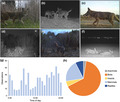

## 1 INTRODUCTION

The COVID‐19 pandemic dramatically altered teaching and learning worldwide, beginning with an abrupt shift to online instruction in spring 2020, with many schools and universities continuing to deliver classes remotely into 2022. This transition to online classes continues to pose a significant challenge for students and educators (Adedoyin & Soykan, 2020), particularly for laboratory classes aimed at providing students with hands‐on research training and for ecology courses that typically involve field‐based activities (Harris et al., 2020; Richter et al., 2021).

The sudden change in human activity due to lockdowns and social distancing also impacted human‐wildlife interactions, with increased sightings of large carnivores reported in many cities (Silva‐Rodríguez et al., 2021; Wilmers et al., 2021; Zellmer et al., 2020). In Los Angeles, for example, there were reports of coyotes and other animals “reclaiming” the city (Sahagun, 2020). It is unclear whether the increased sightings of urban wildlife in Los Angeles and elsewhere were due to pandemic‐induced changes in animal activity or simply greater public attention (Zellmer et al., 2020), but regardless of the underlying causes, the effect of the pandemic on ecological relationships between humans and urban wildlife represents an exciting opportunity for both research and education (Montgomery et al., 2021; Roll et al., 2021; Rutz et al., 2020).

In spring 2021, I aimed to take advantage of this unique situation to implement two pedagogical approaches into an online ecology laboratory course at California State University Dominguez Hills (CSUDH): (1) engaging students in authentic course‐based ecological research and (2) fostering a “sense of place” by centering research experiences on urban wildlife on our university campus. I designed two course‐based research projects focused on evaluating the ecology of urban coyotes (*Canis latrans*), of which there were increased reports during 2020 when the campus was largely depopulated. Students used their research to develop a formal monitoring and management plan for urban wildlife on campus and to create posters to educate the public, providing useful lessons in applied ecology. My goal through these initiatives was to encourage active learning, foster the development of research skills, and inspire students to view themselves as ecologists. While the pandemic necessitated many practical and pedagogical shifts for higher education, it may also have provided important lessons for how research and place‐based learning can be better integrated into undergraduate curricula, particularly at urban, primarily undergraduate universities.

### 1.1 The benefits and challenges of course‐based research and place‐based learning

Actively participating in research can positively impact learning outcomes and enhance the professional development of students in Science, Technology, Engineering, and Mathematics (STEM) (Linn et al., 2015; Lopatto, 2007; Seymour et al., 2004), including in ecology and evolution (Awad & Brown, 2021; Emery et al., 2019). These opportunities may be especially important for students of color, first‐generation college students, and those from communities that continue to be underrepresented and underserved in the sciences (Li & Koedel, 2017; Miriti, 2020; Wanelik et al., 2020), providing them with a potential pathway into STEM careers (Awad & Brown, 2021; Carpi et al., 2017; Hernandez et al., 2018; Lopatto, 2007). In ecology, field‐based research experiences are considered a formative “rite of passage” that allow students to explore ecological concepts in the “real world,” and these opportunities may be especially important for promoting diversity and inclusion in a field that has alarmingly low numbers of underrepresented minorities (Bowser & Cid, 2021; Morales et al., 2020).

Oftentimes, however, such experiences are restricted to working in a research lab under the guidance of a faculty mentor or participation in short‐term research experiences (e.g., summer research experiences for undergraduates). The limited availability and structure of these opportunities may pose significant barriers to the very students that could benefit from them the most, thereby perpetuating existing inequities (Bangera & Brownell, 2014; Morales et al., 2020). Field‐based ecological research programs, for example, may be inaccessible to many students due to financial, social, cultural, or physical barriers, and issues related to gender, ethnicity, race, and identity may prevent some students from participating. A greater incorporation of inquiry‐based research activities directly into required undergraduate coursework represents an important solution for overcoming these challenges and increasing access to the tremendous benefits of engaging in research. Course‐based undergraduate research experiences (CUREs), where students are actively engaged in authentic research in the classroom, are increasingly recognized in biology and other fields as a high‐impact learning activity (Dolan, 2016; Wei & Woodin, 2011). In addition to improving learning outcomes, CUREs may also make scientific research more accessible for students from underrepresented and underserved communities (Bangera & Brownell, 2014).

Place‐based education is another well‐established pedagogical approach where student learning is centered within the context of their own community, physically and culturally (Gruenewald & Smith, 2014). This can be a particularly useful approach in ecology; the local ecosystems—the forests, grasslands, shrublands, and watersheds—that students inhabit become the classroom in which students explore how species interact and respond to the environment (Billick & Price, 2019). By emphasizing a “sense of place” explicitly during teaching, students are encouraged to view key concepts through the lens of their own experience or to ignite a new way of viewing their environment (Semken & Freeman, 2008). An ecologically‐informed sense of place can be instrumental in fostering student engagement and promoting diversity and inclusivity in ecology, conservation, and environmental studies (Bailey et al., 2020; Kudryavtsev et al., 2012). But what about students at campuses in highly urbanized areas? How well do we, as educators, make use of the ecology of cities in our teaching, and how well do we position ourselves to learn from our own students who are experienced naturalists in their own urban environments? By “locating learning” in urban ecosystems, instructors in ecology may improve their ability to capitalize on the cultural strengths of students at urban campuses (Chávez & Longerbeam, 2016). Such an approach has great potential for nurturing a sense of place, enhancing learning outcomes, inspiring environmental stewardship, and facilitating the shift of ecological concepts and theory from the abstract to the concrete (Barnett et al., 2006; Kudryavtsev et al., 2012; Russ et al., 2015).

## 2 CURRICULUM DESIGN AND AIMS

In this paper, I describe the design, implementation, and outcomes of an upper‐level undergraduate laboratory course in ecology in which students were actively engaged in CUREs exploring the ecology of urban wildlife on our campus remotely during the COVID‐19 pandemic, specifically urban coyotes. Students’ research efforts provided an interesting opportunity to evaluate how the pandemic may have impacted campus wildlife, which has important implications for long‐term coexistence and management strategies. My hope is that this teaching approach and the activities described serve as a useful model for other instructors of undergraduate laboratory courses in ecology, particularly those at urban campuses.

### 2.1 Course design

**FIGURE 1:**
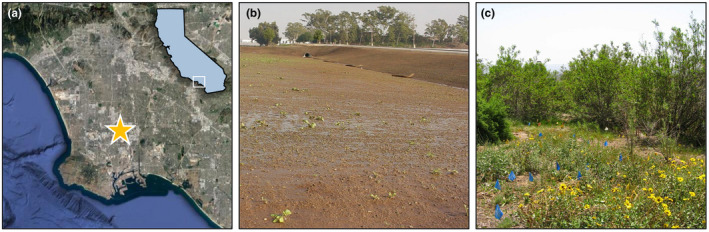
Location of the California State University, Dominguez Hill campus in Carson, California (image from Google Maps; https://www.google.com/maps), in the highly urbanized Los Angeles basin in southern California (a). The Heritage Creek Nature Preserve was established in 2005 (b) following the construction of a parking lot on previously undeveloped land. Following construction, the site was restored with native vegetation (c), with the goal of creating a natural “living laboratory” for students and faculty. The preserve was used as a study site for student research projects, including as a location for several of the camera traps and the collection site for coyote scat samples. Photographs by Constance Vadheim

In spring 2021, I incorporated these activities into an upper‐level ecology laboratory class with 25 students enrolled at CSUDH. This is a one‐unit course that accompanies a three‐unit lecture. CSUDH is a primarily undergraduate university located approximately 20 kilometers south of downtown, Los Angeles (Figure 1a). The university is one of the most ethnically and economically diverse in the United States, with a high proportion of first‐generation college students (CSUDH, 2022; U.S. News, 2020). Multiple sightings of coyotes were reported during a time when the campus was largely vacated due to stay‐at‐home orders and a shift to online instruction. While coyotes have long been known to utilize the university campus, concerns arose about potential risks to the campus community. I used this opportunity to create a student‐led, service‐learning project aimed at obtaining qualitative and quantitative data on urban coyotes and other wildlife while providing students with an authentic inquiry‐based research experience, albeit remotely.

Students collected and analysed data, interviewed experts in urban coyote ecology, completed literature searches, and synthesized their findings in reports and poster presentations. The two primary research activities students completed included (1) the analysis and synthesis of data from camera traps I installed throughout the CSUDH campus to monitor urban wildlife and (2) the analysis of data from scat samples collected on campus to evaluate different the food sources utilized by coyotes on campus. Students also completed a final group project (3) using the information and data generated throughout the semester to develop a monitoring and management plan for urban coyotes on campus. Finally, (4) students created informational posters aimed at educating the public on the ecology of urban coyotes.

### 2.2 Learning goals

Student research projects completed throughout the semester allowed students to explore several key questions in order to learn and apply ecological concepts:

- What is biodiversity and how can we quantify it?
- How do species interact with their environment?
- What impacts do humans have on species and ecosystems, including urban ecosystems?
- How can ecological data be used to guide land management, conservation, and coexistence with urban wildlife?

### 2.3 Skill‐building

In addition to facilitating the learning of ecological concepts, this course aimed to improve students’ abilities and scientific literacy, including:

- Reading and critically evaluating research papers.
- Formulating testable hypotheses.
- Gathering and synthesizing ecological data.
- Displaying and interpreting ecological data in tables and graphs.
- Communicating research outcomes, including in a written research‐article format.

This course also provided students with an opportunity to gain experience in several practical ecological research skills including:

- Species identification.
- Working with ecological data from camera traps and scat dissections.
- Coding and plotting using RStudio.

### 2.4 Online teaching & research: Platforms & tools

I used multiple online platforms and websites for course delivery and to facilitate class discussions, conduct research, and foster a sense of community during remote instruction. Zoom (Zoom Video Communications, San Jose, California) was used to deliver presentations and course materials, demonstrate the use of other platforms and skills via screensharing, hold class discussions, and for students to meet in smaller groups using the Breakout Room function. The communication platform Slack (Slack Technologies, Vancouver, British Columbia) was used for class‐wide communication and direct messaging between students for group work. Dropbox (Dropbox Inc., San Francisco, California) was used to store and view camera trap images, and Google Drive and Google Sheets (Alphabet Inc., Mountain View, California) were used to share files and for data entry and management, respectively. The website iNaturalist (https://www.inaturalist.org/) was used by students to assist with species identification using images and distribution maps available on the site. RStudio Cloud (RStudio, PBC, Boston, Massachusetts; https://rstudio.cloud/) was used for data analysis and plotting. I used TechSmith Knowmia (TechSmith Corporation, Okemos, Michigan) to upload recorded presentations, class meetings, and tutorials for student viewing. Finally, the learning management system Blackboard (Blackboard Inc., Reston, Virginia) was used to make course content and assignments available to students and for grading.

### 2.5 Student research and writing process

**FIGURE 2:**
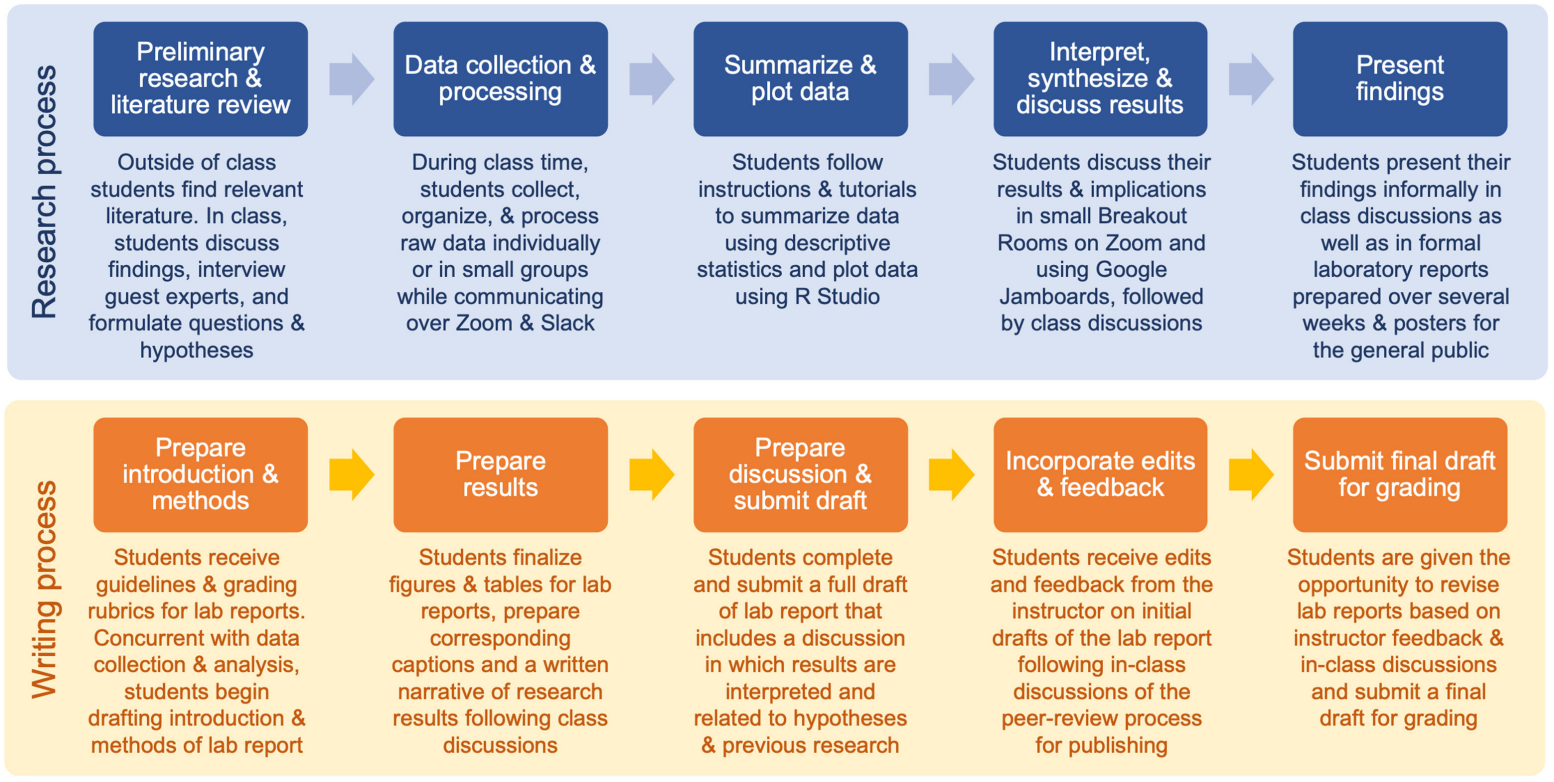
Outline of student research and writing activities for each of the multi‐week laboratory modules. Students gathered preliminary background information relevant to the projects prior to class, which was further developed during in‐class discussions and interviews with guest experts. Students then collected and organized raw data, and generated descriptive statistics, tables, and graphs using RStudio during live class sessions, with instructions and tutorials provided as a guide. Students interpreted and discussed results in small groups and as an entire class and then presented their results in formal laboratory reports, and in posters aimed at educating the public on urban coyotes. Preparation of the laboratory reports was done concurrently with data collection and analysis. Students completed each section of the report (Introduction, Methods, Results, Discussion, and Literature Cited) in stages in an iterative process, in which they were provided individualized feedback and edits before submitting a final draft for grading

For both research projects, students followed a multi‐week research and writing process culminating in formal laboratory reports (Figure 2). The research process required students to individually gather preliminary background information prior to class, including primary research articles, review papers, news articles, and other sources. This information was further developed during in‐class discussions and interviews with guest speakers who were experts on the topics being explored (i.e., urban coyote diet, behavior, and ecology). Students used this information to generate research questions and hypotheses for each of the projects. Students collected (over multiple weeks in the case of the camera trap monitoring) and organized raw data, and generated descriptive statistics, tables, and graphs using RStudio during live class sessions, with instructions and tutorials provided as a guide. Students interpreted and discussed results in small groups (using Breakout Rooms on Zoom) and as an entire class in preparation for presenting these in written reports and posters aimed at educating the public about urban coyotes on campus. Preparation of the laboratory reports was done concurrently with data collection and analysis. Students completed each section of the report (Introduction, Methods, Results, Discussion, and Literature Cited) in stages in a scaffolded process in which they were provided individualized feedback and edits before submitting a final draft for grading.

## 3 PROJECT 1: CAMERA TRAP MONITORING

### 3.1 Project aims

In the first project, students collected and analysed quantitative and qualitative data on urban wildlife (including coyotes) using images captured from camera traps I installed throughout our university campus. The objective of the project was to allow students to gain experience in species identification, quantifying urban biodiversity, and evaluating the activity and behavior of urban coyotes and other animal species.

### 3.2 Methods

In January 2021, camera traps (Bushnell Trophy Cam Trail Cameras; Bushnell Corporation, Overland Park, Kansas) were installed at nine locations throughout the CSUDH campus, including several cameras in the Heritage Creek Nature Preserve (Figure 1b,c). These devices are motion‐sensor cameras capable of capturing digital images in light and dark conditions when triggered by movement in the field of view, with images stored on removable SD memory cards. Cameras were set to the highest degree of detection sensitivity with a delay of 60 s between images once triggered. I downloaded data from each camera every 1–2 weeks and retained all images that contained wildlife observations. Images were uploaded to DropBox, and students reviewed and analysed all images over the course of several weeks throughout the semester. For each image, students identified the species present using online resources (including iNaturalist) and recorded the date, location, and time. These data were compiled into a single spreadsheet for analysis. Students created graphs of the frequency of wildlife sightings (by species and for coyotes based on time of day) using statistical software (RStudio Cloud). In addition to quantitative data, students also interpreted individual images in regard to what ecological information we could gain (e.g., number of unique individuals, age, breeding, behavior, activity, and intra‐ and interspecific interactions).

### 3.3 Outcomes

**FIGURE 3:**
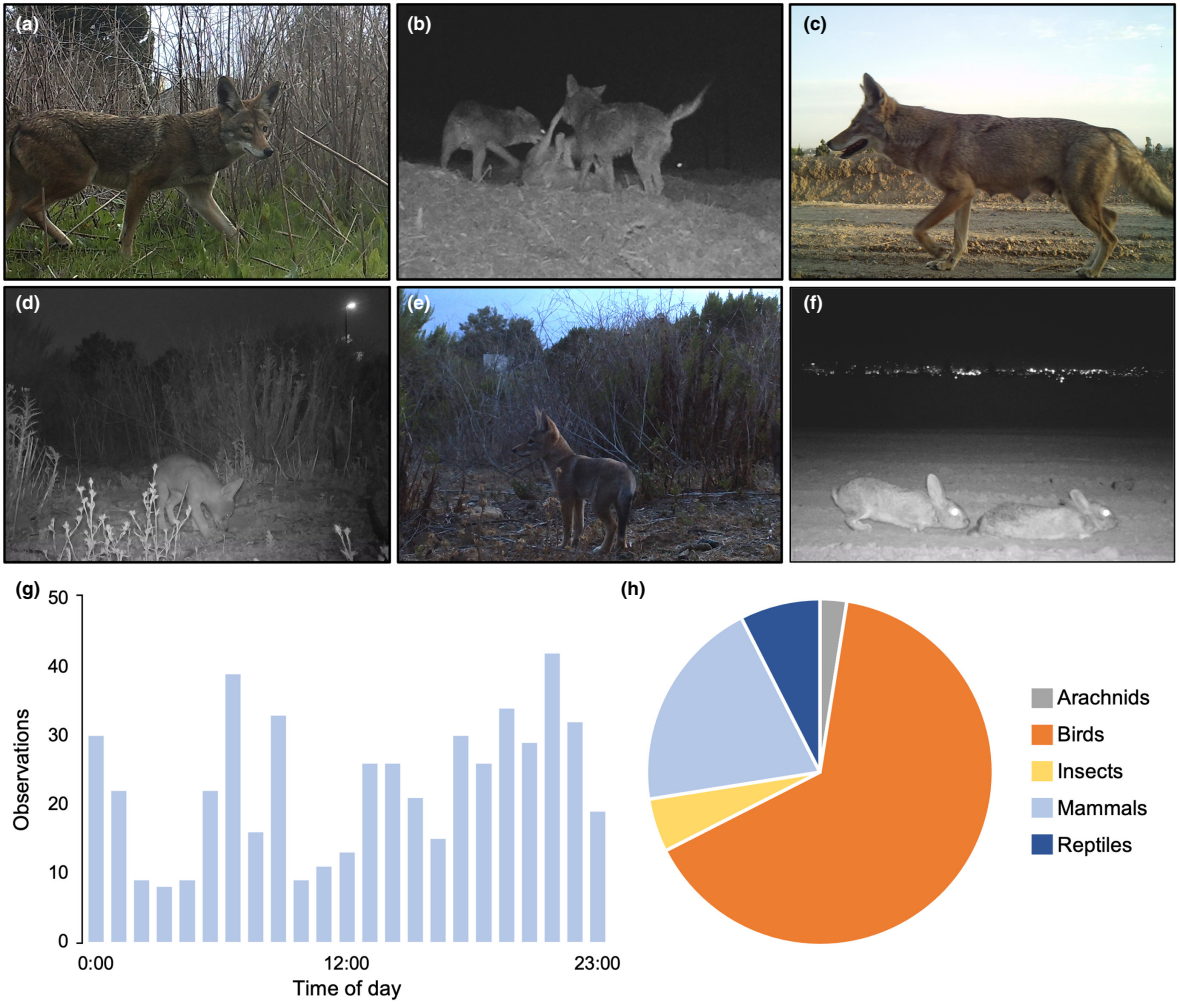
Results of the student‐led camera trap study. Camera traps captured over 400 images of wildlife, which were analysed by students, who identified the species present and recorded the time and location of all sightings. Students were tasked with interpreting the different behaviors exhibited by animals in the captured images, such as hunting, foraging, or breeding. A primary focus of the project was the ecology of urban coyotes (*Canis latrans*; a‐e), which were observed at all locations. Images shown include a frequently observed female in the campus Wetland Preserve (a), a group of three coyotes interacting along a campus roadway (b), a visibly nursing female observed early spring, and her litter of pups observed throughout late spring in the Heritage Creek Nature Preserve (d‐e). The most abundant species recorded by students was the desert cottontail, *Sylvilagus audubonii* (f). Based on student‐generated data and graphs, coyotes appeared to be active at all hours of the day but were most active in the evening and morning (g). In total, students identified 40 different species (across five taxonomic classes) from camera trap images throughout the semester, which included one species of spider, 26 birds, eight mammals, two insects, and three reptiles (h)

The camera trap study provided students with the opportunity to observe urban wildlife on our campus and evaluate animal biodiversity and activity. Students identified a diversity of urban wildlife across the CSUDH campus (Figure 3). The most frequently observed animal species was the desert cottontail (*Sylvilagus audubonii*), the most common species of rabbit in southern California, followed by coyotes (*Canis latrans*), and species of rats (*Rattus spp*.). Other species observed included racoons, opossums, and a variety of bird species. Students explored the frequency of coyote observations by the time of day in order to determine whether there were periods of time when they were more active on campus. Based on the current data analysed, students concluded that coyotes are active at all times of day (Figure 3), but more activity was observed in the evenings and early morning hours.

Students also described ecological information by interpreting individual images (Figure 3). For example, students identified six separate adult coyotes present on campus based on their unique features (e.g., size, coloration, and morphological traits). The most adult individuals observed at a single time were three (Figure 3b). This group included a younger female and older male (with mange) that were frequently sighted together moving through Heritage Creek and are likely a mated pair, as coyotes mate for life (Hennessy et al., 2012). Other transient individuals were also sighted throughout spring 2021 less frequently, including a yearling (born in the previous year) and an adult easily identified by a missing/deformed paw. These data showed that a number of coyote individuals utilize the campus grounds, and that some (e.g., the mated pair) appear to spend a large percentage of their time on campus. Images collected throughout spring 2021 also showed that the mated pair were actively breeding on or near campus. Students observed the female visibly pregnant and nursing in photographs (Figure 3c), and in April 2021, observed the first images of her litter of pups (Figure 3d,e). Students observed three coyote pups in total throughout late spring. Students also reported other ecological interactions, such as coyotes hunting and feeding, rabbits and birds foraging, and rabbits breeding (Figure 3f).

Students presented their results in a formal laboratory report (3–5 single‐spaced pages in length) formatted in a typical research‐article style (i.e., Introduction, Methods, Results, Discussion, and Literature Cited). The report was prepared in a scaffolded and iterative process (Figure 2), where each section was completed and submitted separately, discussed in class, and reviewed and commented on by me, culminating in the final report that was submitted for grading. I provided students with an outline of expectations and a corresponding grading rubric. For the research results, students were required to include two graphs created using RStudio and corresponding captions displaying the total number of observations for each species identified, and a histogram of coyote observation by the time of day (Figure 3g) and two individual images of their choosing captured by camera traps along with a discussion and interpretation of the ecological information learned from the images.

## 4 PROJECT 2: COYOTE DIET ANALYSIS

### 4.1 Project aims

In a second project, students evaluated the different food sources utilized by coyotes on campus through an analysis of scat samples. We were particularly interested in the proportion of anthropogenic items (i.e., trash) in scat samples, as previous research had demonstrated urban coyotes in Los Angeles consume a high degree of human‐sourced food items (Larson et al., 2020). Students hypothesized that scat samples would contain a variety of food items given their omnivorous diet. They also predicted that the shutdown of campus during the COVID‐19 pandemic may have resulted in reduced amounts of human‐sourced food items in coyote diets compared with previous research.

### 4.2 Methods

**FIGURE 4:**
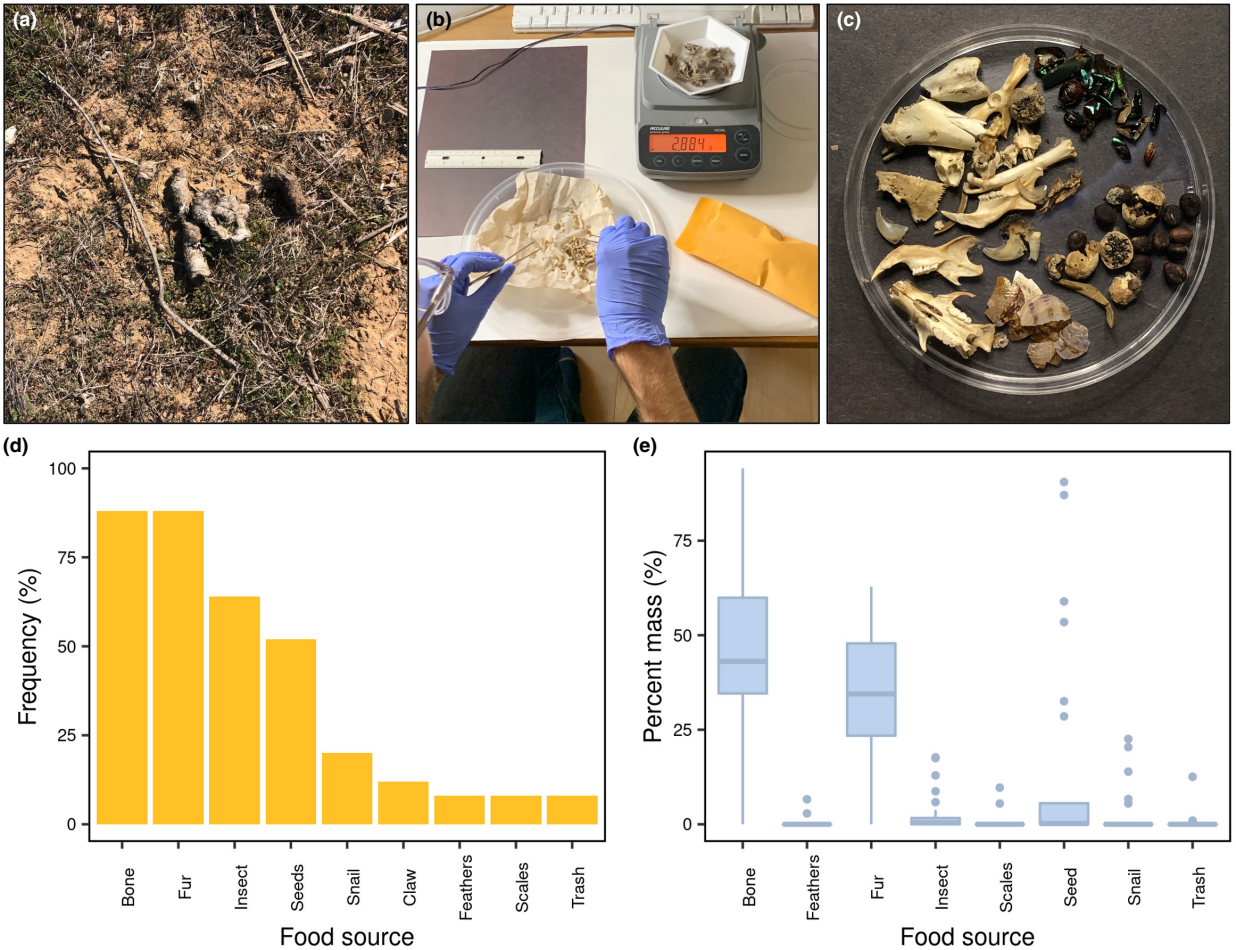
Results from coyote scat data analysed by students. Coyote scat samples were visually identified and collected from Heritage Creek Nature Preserve (a), sterilized, washed, and dissected by hand (b) and then separated into different food sources (c) including anthropogenic sources (i.e., trash), bones, fur, insects, mollusks (snail shells), and plant seeds. Students summarized data using descriptive statistics and plotted the percent frequency of the different food sources identified in scat samples in a bar graph (d) and the percent mass of each food source using boxplots (e) in R Studio. Boxplots display the minimum, maximum, median, and interquartile range

To understand the different food sources utilized by coyotes on campus, students analysed scat samples (*n* = 25) collected in and around Heritage Creek in January 2021 (Figures 1b,c, and 4a). Prior to class, I collected fresh scat samples over a one‐month period and stored samples in a freezer. Samples were dried in a drying oven (48 h at 70°C) and cleaned and dissected by hand (Figure 4b). I separated samples into different food sources including anthropogenic sources (i.e., trash), bones, fur, insects, mollusks, and plant seeds based on visual identification (Figure 4). For each sample, I weighed each category of food item and created a spreadsheet of all raw data for student analyses. In class, students were provided an overview of methods used to collect data and shown images of the different items dissected from samples. Students then used the raw data provided to calculate the percent mass and percent frequency for each food item category. Using RStudio, students created tables of summary statistics and graphs depicting frequency (Figure 4d) and percent mass (Figure 4e) data.

### 4.3 Outcomes

Analysis of scat samples provided students with insight into the different food sources utilized by coyotes on our campus. Based on the high percentage of bone and fur contained in the samples, students concluded that small mammals such as rabbits and rodents represent the major food source for coyotes; these were present in the highest percentage of samples and accounted for the largest proportion of mass (Figure 4d,e). Seeds and insects, while a low proportion of sample mass, were also very frequently observed. Most surprisingly to students, anthropogenic food items (e.g., pieces of trash) were found in only a small percentage of samples, indicating that during the study period, human‐sourced food items were not a major component of coyote diets (based on our limited sampling). Students hypothesized that this was due to the low presence of humans on campus during the COVID‐19 pandemic. Students discussed how the data analysed showed there may be sufficient natural resources on campus (including plants and animals) to sustain urban coyotes. During class discussions, students also noted that coyotes may be performing an important ecosystem service for the campus by controlling rodent pests.

As with the previously described project, students prepared their results in a formal laboratory report, following the guidelines and grading rubric provided. This was again completed in a scaffolded process, where students completed each section (i.e., Introduction, Methods, Results, and Discussion) over the course of several weeks and were tasked with improving and expanding upon these drafts based on class discussions and individualized feedback provided (Figure 2).

## 5 PROJECT 3: COYOTE MONITORING & MANAGEMENT PLAN

At the end of the semester, students worked in small groups (five students per group) to develop a coyote management and monitoring plan for our campus using the information and data they had gathered. The purpose of this exercise was to encourage students to appreciate the importance of applied ecology and how ecological data can be used to guide solutions to “real‐world” problems. In these papers, students were asked to generate recommendations for several specific points: (1) monitoring wildlife activity and behavior; (2) evaluating coyote diet; (3) community education and public outreach; (4) management of campus grounds; and (5) coyote hazing and removal. The documents prepared by students contained a variety of insightful recommendations for the management and continued monitoring of coyotes on campus. However, recurring themes in all of the management plans were the sentiment that efforts should focus on “coexisting” with urban wildlife, the importance of educating the campus community on how to respond when encountering coyotes, and how to mitigate the risk of human‐coyote conflicts.

## 6 PROJECT 4: EDUCATIONAL POSTERS ON URBAN COYOTES

**FIGURE 5:**
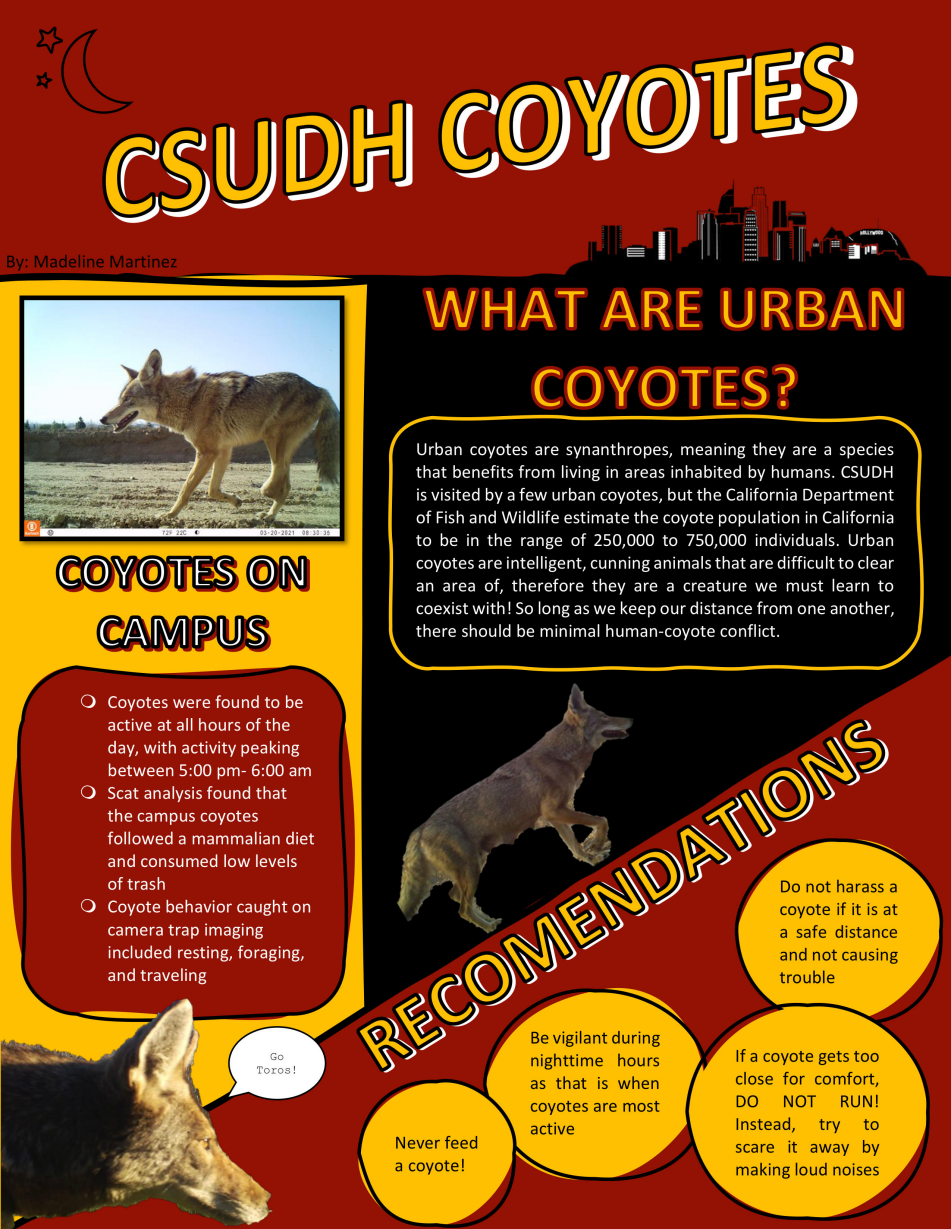
Example of student poster assignment aimed at educating the public and campus community about the ecology of urban coyotes and reduce the risk of human–coyote conflicts. Poster by Madeline Martinez (shared with permission)

For the final project of the semester, students were tasked with creating a poster presentation aimed at educating the campus community about urban coyotes (example shown in Figure 5). The goal of this project was to be an exercise in science communication and to allow students to synthesize the information they had learned throughout the semester. These posters were required to include an overview of urban coyote ecology, a discussion of the key results obtained from each of the research projects, and recommendations for safely coexisting with urban coyotes on campus. Students used these posters to provide educational information on the presence of coyotes on campus, coyote activity, and diet, and what to do when encountering coyotes.

## 7 STUDENT ASSESSMENT AND TESTIMONIALS

I made use of student surveys administered the first week of class and again at the end of the semester to evaluate teaching effectiveness and to gauge how the course had influenced students’ perceptions of their research abilities, overall learning experience, views on the field of ecology, and their identity as scientists.

### 7.1 Survey methods

**FIGURE 6:**
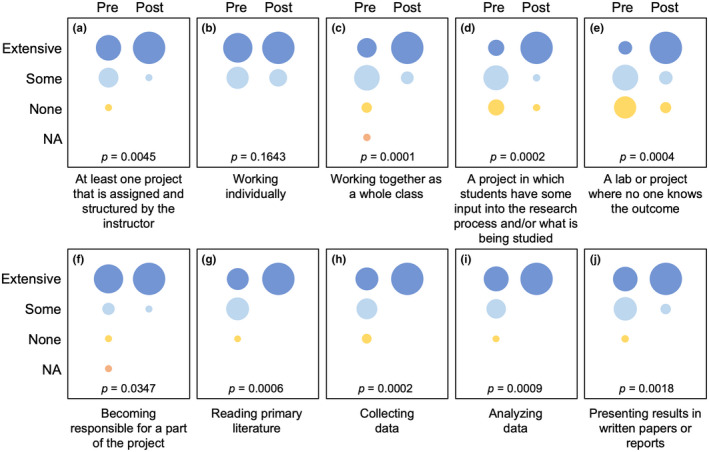
Results of student responses (*n* = 22) from surveys taken at the beginning (pre) and end (post) of the semester. Students were asked at the beginning of the semester to give an estimate of their current level of experience for a variety of research‐related activities and skills (shown below each graph) and at the end of the semester were asked to revisit these questions and provide an estimate of the level of experience gained through the course. Circle sizes are proportional to the number of students who selected a given response (i.e., “extensive,” “some,” “none,” or “NA”). These ordinal, categorical responses were converted to numerical scores, and pre‐ and post‐semester responses were compared using paired *t*‐tests (*p*‐values are shown for each comparison)

**FIGURE 7:**
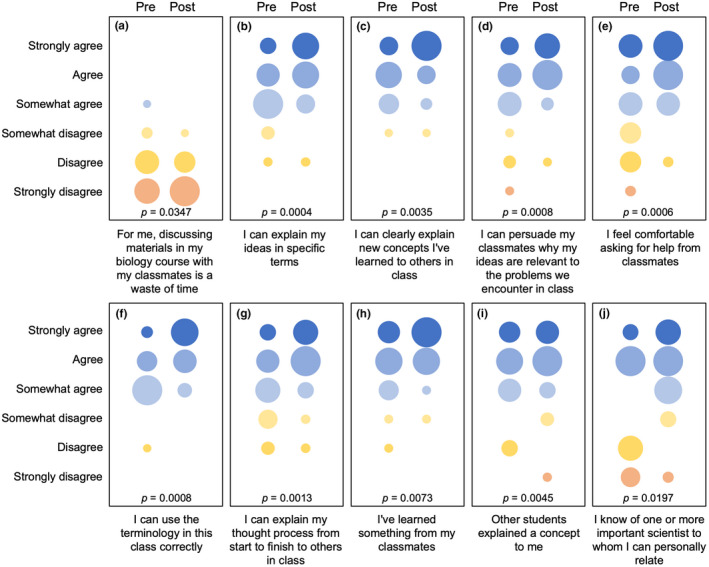
Results of student responses (*n* = 22) from surveys taken at the beginning (pre) and end (post) of the semester. Students were asked the degree to which they agreed (i.e., “strongly agree,” “agree,” “somewhat agree,” “somewhat disagree,” “disagree,” or “strongly disagree”) with a particular statement (shown below each graph) regarding their perceived abilities and learning experience during the semester. Circle sizes are proportional to the number of students who selected a given response. These ordinal, categorical responses were converted to numerical scores, and pre‐ and post‐semester responses were compared using paired t‐tests (*p*‐values are shown for each comparison)

I administered a survey to students at the beginning and end of the semester to evaluate how the course had influenced learning outcomes and experiences (Figures 6 and 7). For questions aimed at understanding students’ perceived level of experience for a given skill or activity, the initial survey asked students to “give an estimate of your current level of experience for…” an activity, and the final survey asked “based on this course, give an estimate of your level of gained experience…” for that same activity (Figure 6), with the options of “NA,” “none,” “some,” and “extensive” given. For other questions, students were asked the degree to which they agreed (i.e., “strongly agree,” “agree,” “somewhat agree,” “somewhat disagree,” “disagree,” or “strongly disagree”) with a particular statement (Figure 7). Of the 25 students enrolled in the class, 22 students completed both the pre‐ and post‐class surveys, and their responses were used for analysis. To analyse survey responses, I converted ordinal categorical responses to a numerical data and used individual paired *t*‐tests to evaluate changes among pre‐ and post‐class responses for each question.

I also administered a separate survey I developed of open‐response questions at the end of the semester to gauge how the course had influenced students’ thoughts on the field of ecology and urban ecology specifically, the role of science in community service, how ecology can guide efforts to coexist with urban wildlife, and how they viewed themselves as scientists (Table 1).

**TABLE 1:** Selected responses to open‐ended survey questions posed to students at the end of the semester regarding their changed perceptions of ecology, the role of ecology in guiding efforts to coexist with urban wildlife and in community service, the field of urban ecology, and how they view themselves as scientists

Question	Responses			
Has this class changed how you think about ecology? If so, how?	I feel like applied ecology is super important because it takes what we learn in the classroom and apply to real‐world problems and come up with real solutions to issues that matter	Yes, I now know how to apply ecological methods to my surroundings and learn how different aspects of my surroundings affect me and other species	It has made me realize how much we use and experience ecology on a daily basis without acknowledging that it is ecology	The involvement and interaction from this class has allowed me to become more interested in the topic of ecology in the real world
How can ecology guide efforts to coexist with urban wildlife?	Based on our research and what I’ve learned in this class, CSUDH should inform more students about our hidden neighbors and bring awareness that they are around	Ecology approaches issues/questions from a scientific view and provides a guide with supporting evidence that can be applied at CSUDH and interpreted elsewhere	Ecology can provide more opportunities for CSUDH to evaluate how our behavior and influences impact urban wildlife around us	CSUDH needs to bring awareness to wildlife on campus and educate students on how to be both respectful and knowledgeable with their space and how to interact with the wildlife on campus
Has this class changed how you view the role of science in community service? If so, how?	At first, I saw ecology as relationships between living and nonliving things with no real significance, but my knowledge through this class has gone deeper in terms of thinking of finding solutions to environmental problems	As an ecologist, I already had an idea of the role ecology played in the world of science. What was new to me is how we are able to utilize ecological studies to managing our landscapes that will be beneficial to humans but also to other life we interact with directly or indirectly	I always thought ecology was interesting but didn't think about how it has implications for the real world or my community until this class like with the camera traps and the coyote management plan	I have always viewed ecology as a community service, but this class has helped in understanding the intersectionality between ecology and other disciplines
Has this class changed the way that you view urban ecosystems or urban ecology? If so, how?	My lower division classes made it seem as though ecology only happened in deserts, rainforests, etc., but this class has shown me that ecology is everywhere! Even right here on campus!	I knew urban ecology was a thing but I didn't realize how much biodiversity we have in urban Los Angeles based on the camera traps on campus	I never had thought about how humans can affect urban ecosystems and animals through social issues like the paper on ecology/evolution and racism	I feel like most people don't appreciate that ecosystems can be anywhere including in cities
Has this class changed the way you see yourself as a scientist? If so, how?	This class has changed greatly the way I see myself as a scientist because I feel like I can make an impact on the world with the techniques and topics we studied during the semester	As an ecology & environmental major I feel a lot more confident in my choice of career. Although the class is virtual, I feel like the skills I have gained are going to help me throughout my entire career	I’m a scientist in training! haven't gotten my B.S., but yes, I feel like anything you want to investigate is valid, and research helps you to become a better scientist	This class has helped me to explore the different approaches I can take for ecological matters and how those approaches may affect the scientific community and the local environment

### 7.2 Survey results

Students showed significant improvements in a variety of skills and strengths as a result of course‐based research activities (Figures 6 and 7). Most students came into the class with some level of experience completing structured research projects (Figure 6a) and working individually (Figure 6b). However, survey responses illustrated that prior to this course, few students had extensive experience working on projects as an entire class (Figure 6c), with projects for which students had input in the research process (Figure 6d), or on research projects for which no one knows the outcome (Figure 6e); students showed significant improvements in their perceived level of experience for each of these. Students also reported significantly higher levels of experience at the end of the semester for being responsible for a part of a research project (Figure 6f), reading primary literature (Figure 6g), collecting (Figure 6h) and analysing (Figure 6i) data, and presenting results in written reports (Figure 6j).

Comparisons of pre‐ and post‐semester survey responses highlighted positive shifts in students’ perceptions of their abilities and comfort levels for different learning activities (Figure 7). At the end of the semester, students showed a greater appreciation for the importance of discussing material with classmates (Figure 7a). Results also showed a positive shift in students’ perceived strengths and comfort level for explaining their ideas in specific terms (Figure 7b), explaining concepts to classmates (Figure 7c), persuading others that their ideas are relevant to problems encountered in class (Figure 7d), asking for help from others (Figure 7e), using terminology encountered in the class correctly (Figure 7f), and explaining their thought process to other students (Figure 7g). At the end of the semester, more students agreed or strongly agreed with the sentiment that they had learned something from their classmates (Figure 7h), and there was an increase in the number of students who felt their classmates had helped explain a concept to them (Figure 7i). Finally, over the course of the semester, there was a significant increase in the number of students who felt that they could personally relate to one or more important scientists (Figure 7j), though it should be noted that this was not universal.

Responses to the open‐ended survey questions posed at the close of the semester also reflected a positive learning experience for many students (Table 1). Multiple students expressed a greater appreciation for the field of ecology (and urban ecology in particular) and its real‐world applications in their responses. Notably, several students expressed that the course had changed the way they viewed urban ecology and that they had gained a greater understanding of urban ecosystems and biodiversity. These views were also expressed by many students during class discussions throughout the semester. For example, one student explained that their lower division classes had given them the impression that ecology was a field of study applicable to only natural areas, but that they now understood that “ecology was everywhere… even on campus.” Multiple responses illustrated that students had gained a greater appreciation for their own local urban ecosystems and the important role of ecology in their own environments and communities.

## 8 LESSONS LEARNED & RECOMMENDATIONS

Training the next generation of ecologists is imperative for meeting the challenges associated with human‐caused environmental change. Many of the skills that students gain through first‐hand research experience are also important for ensuring they are competitive for future career opportunities and graduate school. Enhancing the accessibility of authentic research experiences should therefore be prioritized by educators in the field, especially in light of (and in spite of) the unique challenges imposed by remote learning; many undergraduates have completely missed out on in‐person laboratory courses and research activities since spring 2020. A number of creative solutions and strategies have been proposed for transitioning lab‐based ecology education online (Creech & Shriner, 2020; Harris et al., 2020; Hines et al., 2020; Lashley et al., 2020; Richter et al., 2021). I believe the lessons learned through teaching this class can inform effective teaching strategies in ecology online and once students and instructors return to the classroom, specifically the value of cultivating a sense of place, emphasizing applied ecology and problem‐based learning, and teaching across cultural strengths in ecology classes at urban university campuses.

Students’ research results, management recommendations, and survey responses all illustrate the potential for remote classes to be successful in skill‐building, fostering a sense of community, and conducting meaningful research in ecology. In informal and formal feedback provided, many students expressed how much they enjoyed the research projects (especially working with camera trap data) and reported feeling that they had participated in “real” research, and this enthusiasm was well‐reflected in the quality of the laboratory reports and other assignments prepared throughout the semester. Below, I outline what I believe to be some of the important factors that contributed to the success of this CURE during what was undoubtedly a difficult time for many students taking online laboratory courses (Husky et al., 2020; Lashley et al., 2020; Wester et al., 2021), particularly those students from communities disproportionately impacted by the pandemic in the Los Angeles region (Whitacre et al., 2021).

### 8.1 Cultivating a sense of place: Campus as a living laboratory

One key insight that came out of this course was the valuable role of natural areas on university campuses—especially urban campuses such as ours—as “living laboratories” for student learning. For example, the greatest species richness and coyote activity observed by students using camera traps were in a one‐acre nature preserve on our campus, and many students expressed being pleasantly surprised by the diversity and abundance of wildlife. While students were unable to visit the research sites in‐person, the use of camera traps allowed students to experience the ecology of these areas in a meaningful way despite being online. Even small areas that provide habitat for native species may provide rich opportunities for students to engage with their local species and ecosystems, foster an ecological mindset, advance learning, and improve inclusivity (Bowser & Cid, 2021). As such, creating and maintaining native habitat on university campuses can provide a powerful tool for inquiry‐based learning (Cooke et al., 2021). Furthermore, the close proximity and accessibility of such areas may help educators overcome the logistical challenges and other barriers associated with longer‐distance field trips that could limit the participation of many students.

Using camera traps to monitor wildlife is a well‐established research method in ecology and conservation biology (Burton et al., 2015), and others have highlighted the utility of this method for active learning and CUREs (Edelman & Edelman, 2017; Sorensen et al., 2018), including for the use of monitoring wildlife during the COVID‐19 pandemic (Tripepi & Landberg, 2021). This case study further illustrates the value of this approach for evaluating wildlife activity during the pandemic (Blount et al., 2021) and of utilizing cameras installed on university campuses for student learning. While this may require substantial time and effort on the part of the instructor for deploying cameras and managing images, the benefits for student learning and engagement may be immense. This project allowed students to gain practical skills including the use of camera traps for ecological research, species identification, interpreting animal behavior, quantifying biodiversity, and data analysis. Students also showed a high level of engagement while analysing camera trap images, and many expressed excitement at being able to witness (what several students referred to as) our “hidden neighbors.”

### 8.2 Emphasizing and appreciating the value of urban ecology

Another important lesson that emerged throughout the course was the strong interest of students in urban ecology. In feedback provided by students, a common theme was that students hadn’t previously been given the opportunity to apply ecological concepts and theory to their own local urban ecosystems. One student even wrote in their survey response: “my lower division classes made it seem as though ecology only happened in deserts, rainforests, etc., but this class has shown me that ecology is everywhere! Even right here on campus!” By highlighting urban ecosystems and wildlife in research activities and lessons, instructors at urban universities may better serve their students and capitalize on their existing cultural strengths (Barnett et al., 2006; Chávez & Longerbeam, 2016). For example, most students were unaware of the discipline of urban ecology and had little to no research experience coming into the class. However, when we began our class discussions on urban coyotes, the majority of students had interesting stories of their own encounters with urban wildlife on and off campus that we were able to use to examine ecological concepts, develop research questions, and interpret research results. In this way, I sought to emphasize the cultural strengths of my students as already‐experienced urban naturalists and ecologists of their own environment.

### 8.3 Exploring socio‐ecological connections

I also found students were especially engaged during discussions of socio‐ecological issues as they relate to urban ecosystems. Multiple students reported that their favorite paper read for the course was a recent article by Schell et al. (2020) outlining the ecological and evolutionary consequences of systemic racism in urban environments. I used this paper as a starting point to guide class discussions on how social injustice can impact human and non‐human inhabitants of urban ecosystems and highlight concepts such as the luxury effect, drivers of biodiversity, and urban heat islands as they relate to the city of Los Angeles (Adams et al., 2020; Avolio et al., 2015; Clarke et al., 2013; Vahmani and Ban‐Weiss, 2016), where most students reside. By anchoring ecological theory in the urban ecosystems where students live, educators may be more successful at highlighting the “real‐world” implications, applications, and relevance of ecology, particularly for students that may view (or have been previously taught) nature as something that occurs outside, not within, cities. Given that there is evidence to suggest that underrepresentation in ecology is due largely to the culture of the discipline (Miriti, 2019; Rainey et al., 2018; Taylor, 2018), such efforts could help improve students’ sense of belonging in a field that continues to have low levels of diversity and representation (Bowser & Cid, 2021; Hansen et al., 2018; Kudryavtsev et al., 2012). Related to this, inviting guest scientists from diverse backgrounds into the classroom could be an excellent way to further empower students.

### 8.4 Locating learning: Ecology and the COVID‐19 pandemic

Located learning—connecting content with what is important in students’ lives and current events (Chávez & Longerbeam, 2016)—is an effective pedagogical approach for improving student engagement by highlighting the broader relevance of course content. The projects presented here provided a valuable opportunity for responsive teaching (Robertson et al., 2015) and located learning during the COVID‐19 pandemic. For both research projects, students developed questions and hypotheses within the context of the pandemic; as a class, we asked how changes in human activity and behavior might have influenced the ecology of urban wildlife (Montgomery et al., 2021). For example, while we did not have any pre‐pandemic data on coyote diets, students related their results to previous studies conducted in Los Angeles (Larson et al., 2020) and proposed possible mechanisms contributing to the differences observed (i.e., the lower abundance of garbage on campus during the study period). I also used the ongoing pandemic as an opportunity to introduce students to disease ecology and discuss the eco‐evolutionary factors that may contribute to the emergence of zoonotic diseases and ecologically‐informed strategies to mitigate the risk of future pandemics (Gibb et al., 2020; Roche et al., 2020).

### 8.5 Activating ecological knowledge for problem‐based learning

The original motivation behind these activities was the fact that members of our campus community expressed growing concern about the safety risk posed by coyotes, possibly due to increased coyote sightings early in the pandemic (Sahagun, 2020). In addition to conducting research, students were tasked with applying the information they gathered to generate recommendations for coexisting with urban wildlife and mitigating the risk of human‐coyote conflicts. Actionable solutions proposed by students included educating the campus community about the presence of coyotes and what to do when encountering these animals, securing waste bins to prevent animal access, recommending that dogs be kept on‐leash, and posting signage in areas where coyotes are known to frequent on campus. The class also engaged in lively discussions on the potential pros and cons of coyote hazing—of which there is limited scientific support for (Bonnell & Breck, 2017)—including with a guest scientist with expertise in coyote ecology and human‐wildlife interactions. Laboratory activities therefore represented an excellent example of problem‐based learning, which has been demonstrated to improve engagement, knowledge acquisition and retention, critical thinking, and confidence (Burrow, 2018; Hung et al., 2008). Feedback provided throughout the semester supported the efficacy of such an approach, and students expressed their appreciation of the fact that course activities were being used to generate useful information for our university.

### 8.6 Recommendations for success

I hope the activities and approaches described in this paper serve as a useful model for instructors of undergraduate ecology courses (especially those at urban university campuses) and add to existing suggestions for activating students’ ecological knowledge even during online instruction (Hines et al., 2020). I believe the factors that contributed to the success of this online course and the high level of student engagement—namely emphasizing applied urban ecology, a sense of place, and problem‐based learning—can add to the value of face‐to‐face CUREs for student learning. Indeed, I intend to continue using the laboratory modules developed when teaching this class in‐person (with the added benefit of allowing students to participate even more directly in the research process).

For similar CUREs to be successful, I see several core components. First, grounding projects in local ecosystems and real‐world research questions can be key in driving student engagement. Not all campuses will have similar issues with human‐wildlife conflicts as described in this paper, but educators could task students with quantifying animal biodiversity and behavior. This information could then be used by students to develop similar wildlife monitoring and management plans tailored to their own campuses. This obviously requires investment in camera traps, but such projects could be successful with a fewer number of cameras than used here. The installation and management of camera traps typically required multiple hours per week, which could pose a major constraint for some instructors. For in‐person classes, students may be able to assist with this process and reduce the time required by the instructor. The management of camera traps is also an excellent opportunity for student research assistants; since teaching this class I have hired multiple students to assist with this project, and this could be another useful solution for implementing such a project. If funding is limited, many universities offer students credit for research, which could be another more affordable option.

Second, the scaffolded approach to completing laboratory reports appears to have greatly contributed to student success, improved writing, and synthesis of information. I highly recommend working with students to develop lab reports in a research‐article format over multiple weeks. For larger classes, the individualized feedback I provided my students may not be possible. However, discussing the writing process as an entire class and utilizing peer review and editing are possible alternatives that would require less time for instructors. Using dedicated class time to analyse, review, and discuss research results also allowed students to better understand key concepts and implications. The activities outlined here served as a useful introduction to RStudio for my students, and these could be further strengthened by the incorporation of statistical analyses appropriate for upper‐level ecology students.

Institutional support may also be important for the success of such projects. Many laboratory classes are designed to match the weekly content of accompanying lecture sections, but this is not as feasible when implementing multi‐week research modules as opposed to separate weekly activities. Therefore, course redesigns will likely require buy‐in from multiple instructors or entire departments. These types of laboratory courses may also be more difficult to implement when there is a high degree of turnover in instructors (e.g., courses taught by adjunct faculty or teaching assistants). Designing CUREs and initially implementing them may be a greater workload than traditional laboratory classes, but ideally over time these will require less time and effort to teach. Using campuses as a “living laboratory” may also require university resources and support in order to create and maintain areas that will meet the needs of instructors. For example, the creation of native plant gardens or nature preserves may not be possible at all campuses. However, similar projects could be completed taking advantage of already‐existing gardens or landscaping.

Finally, I encourage instructors to administer pre‐ and post‐laboratory course surveys to students. While this also represents a significant time investment and requires careful planning, this may be instrumental (as evidenced here) in evaluating the success of instructional approaches and identifying factors that may better facilitate student learning.

## AUTHOR CONTRIBUTION

**Justin M. Valliere:** Conceptualization (lead); Formal analysis (lead); Methodology (lead); Project administration (lead); Visualization (lead); Writing – original draft (lead); Writing – review & editing (lead).

## CONFLICT OF INTEREST

The author has no conflicts of interest to report.

## Data Availability

Student survey data and scat dissection and wildlife observation data used to create examples of student research products: Dryad data repository https://doi.org/10.5061/dryad.qz612jmhz
